# Complete Genome Sequence of the *Edwardsiella ictaluri*-Specific Bacteriophage PEi21, Isolated from River Water in Japan

**DOI:** 10.1128/genomeA.00228-14

**Published:** 2014-04-03

**Authors:** Motoshige Yasuike, Wataru Kai, Yoji Nakamura, Atushi Fujiwara, Yasuhiko Kawato, Ebtsam Sayed Hassan, Mahmoud Mostafa Mahmoud, Satoshi Nagai, Takanori Kobayashi, Mitsuru Ototake, Toshihiro Nakai

**Affiliations:** a Research Center for Aquatic Genomics, National Research Institute of Fisheries Science, Fisheries Research Agency, Yokohama, Japan; b Diagnosis and Training Center for Fish Diseases, National Research Institute of Aquaculture, Fisheries Research Agency, Mie, Japan; c Faculty of Veterinary Medicine, Assiut University, Assiut, Egypt; d Graduate School of Biosphere Science, Hiroshima University, Higashihiroshima, Japan

## Abstract

We present the complete genome sequence for a novel *Edwardsiella ictaluri*-specific bacteriophage, PEi21, isolated from river water in Japan. An initial comparative genome analysis revealed that the phage was closely related to the previously reported *Edwardsiella tarda* phage MSW-3 isolated from a red sea bream farm in Japan.

## GENOME ANNOUNCEMENT

*Edwardsiella ictaluri*, a Gram-negative bacterium, causes enteric septicemia of catfish (ESC), the most serious infectious disease in the channel catfish (*Ictalurus punctatus*) industry in the United States (1). *E. ictaluri* also infects other catfish species and is found in Southeast Asia (2–4). For a long time there had been no records of this bacterium in Japan. However, in 2007, *E. ictaluri* infections of wild ayu (*Plecoglossus altivelis*) were first found in some rivers of Japan (5, 6) and have been continuously observed since then (7).

Three *E. ictaluri*-specific bacteriophages (phages), eiAU, eiDWF, and eiMSLS, have been isolated from catfish aquaculture pounds in the United States and classified within the family *Siphoviridae* (8, 9). Recently, *E. ictaluri*-specific phages have also been isolated from rivers in Japan, in association with *E. ictaluri* infections of ayu (7). These phages exhibited the morphology of the family *Myoviridae* (7). To further characterize the *E. ictaluri* phages isolated from the Japanese rivers, we determined the complete genome sequence of the *E. ictaluri*-specific bacteriophage PEi21.

Whole-genome shotgun sequencing of PEi21 was performed using the Roche 454GS-FLX Titanium sequencing platform. *De novo* assembly of sequence reads was performed using Newbler 2.8. The complete genome sequence was annotated using the Rapid Annotations using Subsystems Technology (RAST) server (10) and BLASTP (11) against the viral sequence database (*E* value threshold of 1E−3).

The complete genome sequence of PEi21 was assembled as a circular contig. The circularly permutated genome showed a 43,378-bp length, with a GC content of 52.6%. The genome contained 71 predicted open reading frames (ORFs), of which 59 encode conserved hypothetical proteins or novel proteins and 12 have a predicted function. A phylogenetic analysis based on portal proteins revealed that PEi21 was closely related to dwarf myoviruses (12). The *E. tarda* phage MSW-3, which was isolated from a seawater sample obtained from a red sea bream (*Pagrus major*) farm in Japan (13), was the closest phage to PEi21, followed by *Klebsiella* phage JD001 (14), Iodobacteriophage φPLPE (15), *Vibrio* phages 138 and CP-T_1_ (12), and *Pectobacterium* phage ZF40 (12). CoreGenes3.5 (16) analysis (with the BLASTP threshold score set at 75) also confirmed these results, showing that PEi21 shared 54 homologous genes with MSW-3, while PEi21 has 40, 28, 31, and 20 homologous genes in common with *Klebsiella* phage JD001, Iodobacteriophage φPLPE, *Vibrio* phages 138 and CP-T1, and *Pectobacterium* phage ZF40, respectively. Thus, it will be interesting to investigate the function of the unique genes among these phage genomes in future studies, which will increase our understanding of the evolution of these phages and their host specificity. Furthermore, this *E. ictaluri* phage genome information provides a novel resource for detection of *E. ictaluri* in natural freshwater, for elucidating the transmission route of *E. ictaluri* (7), and for various applications of phages in the control of ESC and other *E. ictaluri* infections in aquaculture (17).

### Nucleotide sequence accession number.

The complete genome sequence of the *E. ictaluri* phage PEi21 was submitted to DDBJ under the accession number AP013057.
